# Local Injection versus Topical Microneedling of Platelet-Rich Plasma for Androgenetic Alopecia: A Systematic Review

**DOI:** 10.1055/a-2510-5517

**Published:** 2025-03-11

**Authors:** Johannes Albert Biben, Ryan Reinhart, Karina Karina, Kuswan Ambar Pamungkas, Krista Ekaputri, Patricia Marcellina Sadikin

**Affiliations:** 1 Hayandra Clinic, Hayandra Peduli Foundation, Central Jakarta, Indonesia; 2 Tobelo Regional General Hospital, Jl.Landbouw, North Halmahera, North Maluku, Indonesia; 3 HayandraLab., Yayasan Hayandra Peduli, Central Jakarta, Indonesia; 4 Faculty of Medicine, Pembangunan Nasional University, Veteran Jakarta, Indonesia; 5 Stem Cell Study Center, Pembangunan Nasional University Veteran, Jakarta, Indonesia

**Keywords:** platelet-rich plasma, microneedling, androgenetic alopecia

## Abstract

Autologous platelet-rich plasma (PRP) has gained popularity for hair restoration due to its effectiveness and safety. PRP could be administered through direct local injections to the scalp or applied topically with the aid of microneedling therapy. This systematic review aims to elaborate on the effectiveness of PRP administered with syringe injection and topical PRP with microneedling combination for the treatment of androgenetic alopecia (AGA). A literature search was employed through PubMed, Cochrane Central Register of Controlled Trials, Embase, Web of Science, and Scopus. The database was searched using terms and keywords: “platelet-rich plasma” and “microneedling” and “androgenetic alopecia.” Inclusion criteria are human study, patients with AGA, studies that compare PRP with syringe injection and the combination of PRP and microneedling. Exclusion criteria are animal study, review, case reports, or studies on other form of alopecia.

A total of 108 articles found in the database. Title and abstract screening yield 12 articles. After full-text reading three articles were included in the review. A combination of PRP and microneedling appears to yield more superior results than direct syringe injection. Topical PRP and microneedling potentially give better results on AGA cases. Further high-quality studies with uniform protocol are needed to confirm these findings.

**Level of Evidence** I.

## Introduction

Hair beyond its practical function serves as a crucial component of identity; its loss through alopecia impacts quality of life and potentially serves as a risk factor for depression and anxiety by affecting one's perceived attractiveness. 1 2 Among the various forms of alopecia, androgenetic alopecia (AGA) stands out, stemming from a complex interplay of genetic, hormonal, and environmental factors. 3 4

In the pursuit of effective AGA therapies, a myriad of modalities and drug delivery techniques can be found in the literature. Clinicians and researchers alike are on a continuous effort to optimize the treatment outcomes while mitigating the adverse effects. 5 Numerous minimally invasive treatments for the management of AGA have been extensively studied in recent years including mesotherapy, carboxytherapy, light devices, botulinum toxin, microneedling, and platelet-rich plasma (PRP). 6 7

Among these modalities, autologous PRP has emerged as a favored choice for hair restoration due to its proven efficacy and safety profile. 3 The administration of PRP whether through direct local injections or topical application aided by microneedling therapy has garnered considerable attention. 8 9 Some patients and clinicians prefer the combination of microneedling and PRP to local syringe injection, because it might offer synergistic therapeutic effects, better PRP distribution, and less painful procedure. On the other hand, syringe injection assures most of the PRP volume will be delivered into the dermis or subdermis, while some of the topically applied PRP might be left out on the hair and scalp surface. 10 11 12 13 14

This systematic review embarks on an exploration of the efficacy of PRP administered via syringe injection versus the combination of topical PRP with microneedling for AGA treatment. By synthesizing existing evidence, this review aims to aid practitioners and patients with insights to discern the most effective and safest method for delivering PRP therapy in the context of AGA.

## Methods

### Search Strategy

This systematic review was conducted according to the Preferred Reporting Items for Systematic Reviews and Meta-Analyses protocols. 15 16 A literature search was performed on May 15, 2023 using “platelet-rich plasma” and “microneedling” and “androgenetic alopecia” as the keywords. The search terms included related terms in the Medline Medical Subject Heading Library. The literature search was limited to studies only on human subject. PubMed, Cochrane Central Register of Controlled Trials, Embase, Web of Science, and Scopus databases were explored for this systematic review. Systematic reviews with similar topics were scrutinized for potential additional relevant studies.

### Eligibility Criteria

**Fig. 1:**
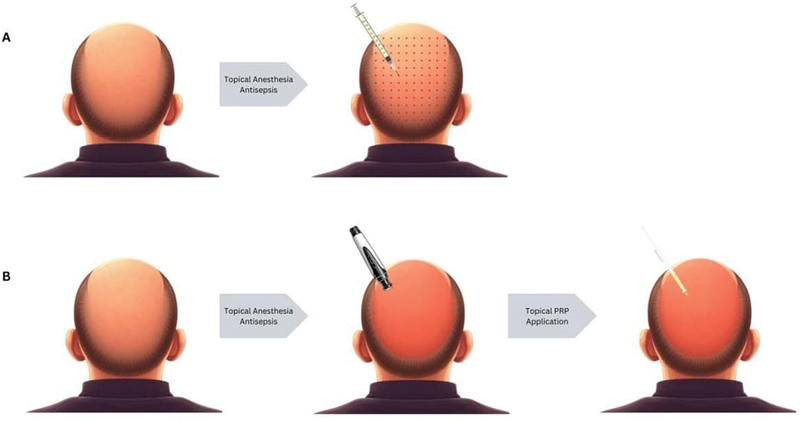
Comparison of local injection ( **A** ) and topical microneedling ( **B** ) of platelet-rich plasma application.

We included studies comparing PRP injection and the combination of microneedling and topical PRP application for the treatment of AGA ( Fig. 1 ). The inclusion criteria for this review were studies on human subjects. Randomized clinical trial, nonrandomized clinical trial (RCT), cohort, and retrospective studies were included in this review. We excluded studies that involved subjects with other form of alopecia unrelated to AGA (i.e., scarring, alopecia areata, autoimmune, or other systemic condition). In vitro study, case series, case reports, and review articles were also excluded.

### Selection Process

Authors (J.A.B., R.R., K.K., P.M.S.) independently assessed the titles and abstract of the studies to identify relevant studies. Full-text reading was performed to select the studies included in this review. 17 Studies selection was based on inclusion and exclusion criteria with consensus between all authors.

### Data Collection, Assessment, and Analysis

Information regarding subject characteristics, therapy protocol, length of follow-up, treatment outcomes, and adverse effects were extracted from the included studies. The quality and bias risk of the studies were evaluated with the Modified Jadad Scale. 18 19

## Results

**Fig. 2:**
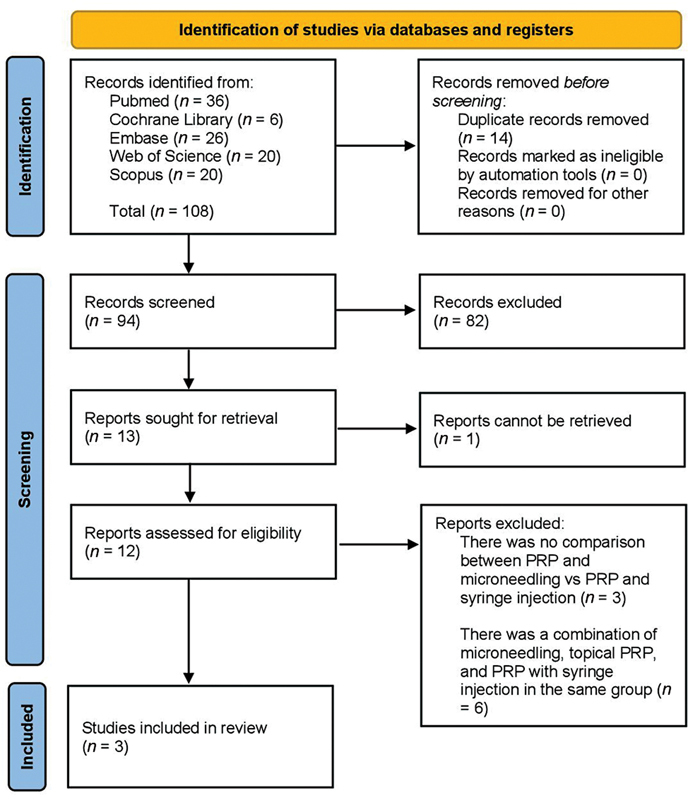
PRISMA flowchart. PRISMA, Preferred Reporting Items for Systematic Reviews and Meta-Analyses.

A literature search in the databases yielded 108 articles. Twelve articles were relevant based on title and abstract screening. Three articles were included in this review after thorough full-text reading ( Fig. 2 ). Three studies were excluded, because they did not compare the efficacy of topical PRP and microneedling with PRP administered by syringe injection. 20 21 22 Six studies were excluded, because they combine PRP injection and microneedling with or without topical PRP application in one treatment group. 23 24 25 26 27 28

All of the studies included in this review were randomized controlled trials. However, there were some limitations in these studies, particularly in the blinding process. Two studies scored 4 on the Modified Jadad Scale, were considered high-quality RCTs, whereas the one that scored 3.5 had slightly lower quality. 19 The quality analysis of those studies was presented in Table 1 . 19 The studies' protocols were elaborated in Table 2 . 12 13 14 In the study conducted by Muhammad et al, the hair pull-test conversion (82.1 vs. 51.9%), hair count increase (24.53 ± 9.49 vs. 17.88 ± 10.15%; *p* = 0.011), and patients' satisfaction improvement parameters were better in the group treated with the combination of PRP and microneedling than conventional PRP injection. 12 Ramadan et al reported that the group treated with the combination of microneedling and PRP demonstrated the highest improvement in hair volume/density by clinical evaluation (78.3 ± 10.6), hair density by dermoscopy (56.3 ± 25.2; *p* < 0.001), and hair diameter by dermoscopy (52.3 ± 34.0; *p* < 0.001), compared with the group treated with PRP injection alone and control group. 13 Ozcan et al also reported better outcomes on the hair count (4.60 ± 9.76 vs. 4.14 ± 8.04; *p* = 0.838) and hair density (8.39 ± 17.78 vs. 7.57 ± 14.71; *p* = 0.833) in the combination group, but it was not statistically significant. However, they found that subjects treated with the combination of topical PRP and microneedling showed significant improvement in the anagen/telogen hair ratio ( *p* < 0.05). 14 The outcomes of the included studies were summarized in Table 3 , whereas the adverse effects were summarized in Table 4 . 12 13 14

**Table 1:** Modified Jadad Scale 19

Study Items	Muhammad 12	Ramadan 13	Ozcan 14
Was the study described as randomized?	+1	+1	+1
Was the method of randomization appropriate?	+1	−1	+1
Was the study described as blinded?	0	0, 5	0
Was the method of blinding appropriate?	0	+1	0
Was there a description of withdrawals and dropouts?	0	0	0
Was there a clear description of the inclusion/exclusion criteria?	+1	+1	+1
Was the method used to assess adverse effects described?	0	+1	0
Was the method of statistical analysis described?	+1	+1	+1
Total score	4	3, 5	4

**Table 2:** Summary of included studies

Study	Total subjects	Intervention	PRP protocol	Injection and microneedling protocol	Additional therapy a	Treatment and follow-up protocol
Muhammad et al 12 (2022)	*n* = 60 Male = 57 Female = 3 Group I = 30 Group II = 30	Group I conventional PRP Group II PRP + microneedling	Volume of blood: 20 mL Anticoagulant: citrate phosphate dextrose with adenine Centrifugation I: 1500 rpm, 15 min Centrifugation II: 4000 rpm, 10 min Platelet activator: yes, with calcium gluconate	Device: dermaroller • Depth: 2 mm • Passes: no data • Injection • Syringe: 1 mL • Needle: no data • Space: no data	No data	3 sessions 1-mo interval Follow-up per 3 mo
Ramadan et al 13 (2020)	*n* = 126 Male = 46 Female = 80 Group I = 42 Group II = 42 Group III = 42	Group I conventional PRP Group II PRP + microneedling Group III control	Volume of blood: 10 mL Anticoagulant: citrate Centrifugation I: 547.82 G, 10 min Platelet activator: no	Device: dermapen • Depth: 2 mm • Passes: 3 times • Injection • Syringe: no data • Needle: no data • Space: no data	Female: topical minoxidil 5% qd, oral spironolactone 100 mg qd Male: topical minoxidil 5% bid, oral finasteride 2.5 mg qd	3–6 sessions 1-mo interval Follow-up • Third month • Sixth month 3 mo after last session
Ozcan et al 14 (2021)	*n* = 62 Male = 62 Female = 0 Group I = 31 Group II = 31	Group I conventional PRP Group II PRP + microneedling	Volume of blood: 10 mL Anticoagulant: citrate Centrifugation I: 2800 G, 8 min Platelet activator: no	Device: dermapen • Depth: 1.5 mm • Passes: no data • Injection • Syringe: 5 mL • Needle: 30 G • Space: per cm ^2^	No additional therapy	4 sessions Interval • First three sessions: 2 wk • Last session 1 mo after the third session. Follow-up: no data

a bid, twice daily; qd, once daily.

**Table 3:** Summary of outcomes

Study	Hair count/volume/density/diameter	Change of anagen/telogen hair	Hair pull test	Patient assessment	Physician assessment
Muhammad et al 12 (2022)	Hair count increase (mean ± SD) Group I: 17.88 ± 10.15% Group II: 24.53 ± 9.49% *p* = 0.011	–	Hair pull test conversion (positive to negative) Group I: 51.9% Group II: 82.1%	Conversion of hair loss perception from “severe” to “mild/moderate” Group I: 73.9% Group II: 88%	–
Ramadan et al 13 (2020)	1. Improvement of hair volume/density by clinical evaluation (mean ± SD) Group I: 64.3 ± 14.3 Group II: 78.3 ± 10.6 Group III: 35.7 ± 14.9 *p* < 0.001 (between groups) *p* = 0.036 (group I vs. II) 2. Improvement of hair density by dermoscopy (mean ± SD) Group I: 16.7 ± 12.8 Group II: 56.3 ± 25.2 Group III: 10.8 ± 8.4 *p* < 0.001 (between groups) *p* < 0.001 (group I vs. II) 3. Improvement of hair diameter by dermoscopy (mean ± SD) Group I: 20.3 ± 16.7 Group II: 52.3 ± 34.0 Group III: 13.5 ± 6.6 *p* < 0.001 (between groups) *p* < 0.001 (group I vs. II)	–	Negative in over 95% of all patients	Satisfaction rate: 88% of all patients	–
Ozcan et al 14 (2021)	1. Improvement of hair count (mean ± SD) Group I: 4.14 ± 8.04 Group II: 4.60 ± 9.76 *p* = 0.838 2. Improvement of hair density (mean ± SD) Group I: 7.57 ± 14.71 Group II: 8.39 ± 17.78 *p* = 0.833	1. Change of anagen hair (mean ± SD) Group I: −6 ± 16.06 Group II: 6.52 ± 19.74 *p* = 0.016 2. Change of telogen hair (mean ± SD) Group I: 6 ± 16.06 Group II: −6.65 ± 19.73 *p* = 0.014	1. Before vs. after in both group I and group II *p* < 0.001 2. After treatment: group I vs. group II *p* = 0.506	Group I vs. group II *p* > 0.05	Group I vs. group II *p* > 0.05

Abbreviations: PRP, platelet-rich plasma; SD, standard deviation.

Note: Group I—PRP injection; Group II—PRP + microneedling; Group III—control.

**Table 4:** Summary of adverse effect

Study	Adverse effect a
Muhammad et al 12 (2022)	The “severe” and “very severe” pain perception level Group I: 40% Grade II: 0%
Ramadan et al 13 (2020)	Group I (23 patients): burning pain, scalp pain, headache Group II: none Group III: none
Ozcan et al 14 (2021)	No data

Abbreviation: PRP, platelet-rich plasma.

a Group I: PRP injection; Group II: PRP + microneedling; Group III: control.

**Fig. 3:**
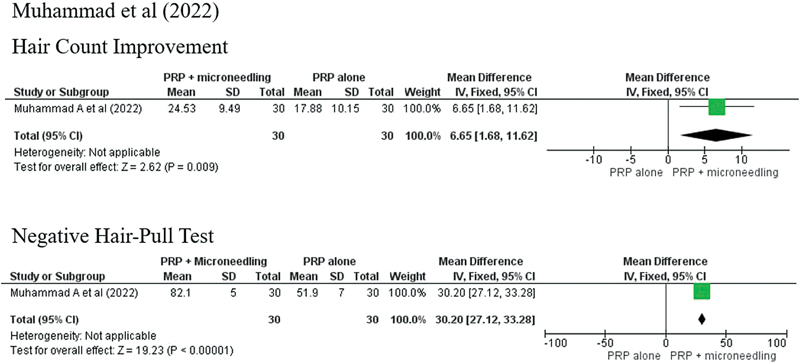
Forest plot Muhammad).

The outcomes of the treatment could not be pooled as there were significant variations in the treatment protocols and outcome parameters. In general, the combination of microneedling and topical PRP produced better results in the improvement of hair count, hair density, hair diameter, and anagen/telogen ratio than subjects treated with PRP injection ( Figs. 3 4 5 ).

**Fig. 4:**
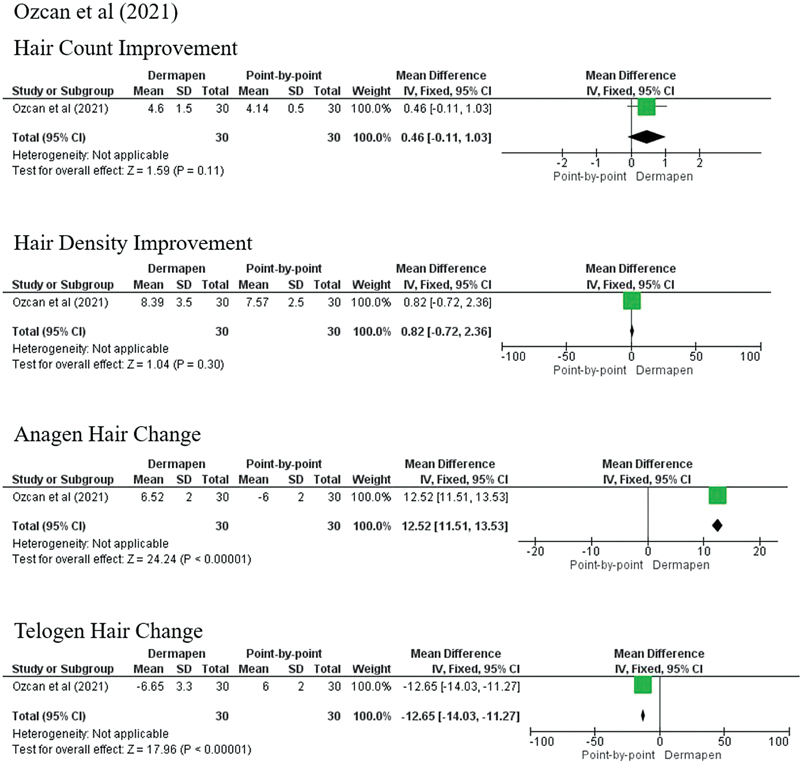
Forest plot Ozcan et al (2021).

**Fig. 5:**
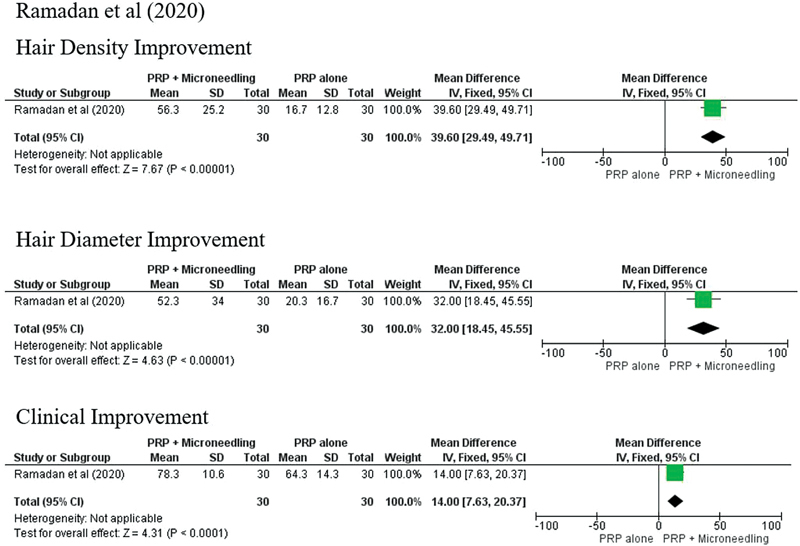
Forest plot Ramadan et al (2020).

Adverse events were reported only in one study, 13 which was 54.7% in the PRP injection group and none were reported in the group combining microneedling with topical PRP. The adverse events reported were burning pain, scalp pain, and headache that disappeared after a few hours with paracetamol medication.

## Discussion

Numerous pieces of literature have supported the use of PRP to treat AGA, as it contains various growth factors and cytokines that enhance the growth and regeneration of hair follicles. 28 29 Research has shown that growth factors can also enhance blood flow and extend the anagen phase of the hair growth cycle, addressing the causes of AGA. Additionally, growth factors aid in activating the differentiation of hair and stem cells, which promotes the formation of new hair follicles. 14 It gained popularity because it is considered an effective, simple, and safe treatment to restore hair growth. 28 30 PRP is usually delivered to the scalp by direct syringe injection throughout the treatment area. 28 30

Microneedling is a minimally invasive procedure involving the use of multiple needles that puncture through the skin to create microchannels. Microneedling is widely recognized for boosting the release of growth factors like vascular endothelial growth factor. It also increases the expression of Wnt3a, β-catenin, and Wnt10b at both the mRNA and protein levels, which contributes to hair growth. 13 Microneedling can be used as a solitary treatment for AGA or in combination with other treatments, including PRP. 31 When combined with PRP, microneedling can also serve as a technique to deliver the topically applied PRP into the hair-bearing skin. Some patients prefer microneedling to syringe injection because it is less painful. 12 Several clinicians also suggested that microneedling results in better PRP distribution to the treated area. 26 27

Some clinicians still performed PRP injection, even though they treated the patient with microneedling technique in the same treatment session. 23 24 25 26 27 28 The reluctance to simply apply the PRP topically during a microneedling session might be caused by concerns about its effectiveness to deliver the PRP into the hair follicle and its surrounding tissue. Some patients and practitioners doubt the efficacy of this combination therapy because a fraction of the PRP volume would be inevitably left out on the skin and hair surface.

The findings in this systematic review showed that the combination of microneedling and topical PRP to treat AGA was superior in several parameters compared with PRP applied by injection technique. The possible explanation for this finding is that the microneedling procedure not only facilitated adequate PRP penetration and even distribution into the scalp dermis, but also produced a follicle-stimulating effect that worked synergistically with the effects of PRP.

The reviewed studies presented a range of outcome parameters in various manners, as outlined in Table 3 , thereby hindering direct comparison among them. Based on the hair count or hair density parameter, all of the included studies reported that the improvement in the combination group is better than the PRP injection group. Muhammad et al and Ramadan et al found a statistically significant difference. 12 13 Ozcan et al identified a significant improvement in hair count and density within each group after treatment with PRP injection and the combination of topical PRP and microneedling. The improvement in hair count and density in the combination group was greater than that with the conventional PRP injection, but it was not statistically significant. 14 Even though Ozcan did not find a significant difference in the hair count or density between the two groups, they reported that the combination of microneedling and topical PRP improved the anagen/telogen hair ratio significantly. The group treated with microneedling and topical PRP exhibited an increase in anagen hair (6.52 ± 19.74) and a decrease in telogen hair (−6.65 ± 19.73). The opposite was found in the group treated with PRP injection. There was an increase in telogen hair (6 ± 16.06) and a decrease in anagen hair (−6 ± 16.06). 14 Patients with AGA experience an increase in telogen hair and a decrease in anagen hair. Thus, a treatment capable of reversing this condition is deemed beneficial for the patients. 14 Ramadan et al reported that subjects treated with the combination of microneedling and topical PRP demonstrated 2.5 and 3.8 times greater improvement in hair diameter compared with the PRP injection group and control group, respectively. 13

The posttreatment hair pull test conducted by Muhammad et al revealed that the group with combination therapy had a higher percentage of conversion to negative hair pull test. 12 On the other hand, Ramadan et al and Ozcan et al found comparable results between the two groups. 13 14 Two studies evaluated patient self-assessment parameters using different tools. Muhammad et al reported superior results in the combination therapy group based on patient assessment parameters, whereas Ozcan found no statistically significant difference between the two treatments on patient's and physician's satisfaction assessment. 12 14

There is some notable variability regarding the methods among the included studies. The volume of blood that was drawn for PRP ranged from 10 to 20 mL. Muhammad et al performed two centrifugation steps and added platelet activator, whereas the other two studies performed one-time centrifugation without platelet activation. 12 13 14 Both activated and inactivated PRP had been proven to be effective to treat AGA based on previous systematic reviews. 28 29 30

Regarding the microneedling procedures, the devices used for microneedling were dermaroller and dermapen with 1.5- to 2-mm needle penetration depth. Hori et al found that the thickness of the epidermis and the hair-bearing dermis on the scalp evolved. 32 The dermis layer got thicker until a certain age (around 35 years old in women and 55 years old in men) before getting thinner. Women over the age of 70 got a second rise of dermal thickness, whereas men did not. The thickness of the epidermis and dermis during that period ranged from 0.04 to 0.07 and 0.8 to 1.5 mm, respectively. 32 Therefore, the needle penetration depth of 1.5 to 2 mm is sufficient to deliver the PRP into the skin layer containing hair follicle, as also demonstrated by Sasaki in their experiment. 20

The spacing of the PRP injection technique only described by Ozcan et al, which was one injection every 1 cm ^2^ . 14 To ensure a good PRP distribution to the treated area, numerous injections need to be performed on the scalp. This might be related to the higher adverse event rate in the PRP injection group as reported by Muhammad et al and Ramadan et al. 12 13 The most commonly reported adverse events are related to pain. Although it is not a serious complication, it is a limiting factor that might negatively impact patient compliance, potentially leading to suboptimal treatment outcomes in the clinical setting. 21 Poor compliance could result in poor treatment outcomes in the clinical setting. The use of microneedling to facilitate PRP treatment demonstrated a reduction in pain for the patients. 11

In summary, all studies agree that the combination of microneedling and topical PRP to treat AGA appears to yield more superior outcomes in several parameters than injected PRP. It also had a better safety profile with almost no reported adverse events. Therefore, it is evident that microneedling could facilitate PRP penetration into the targeted hair follicles with excellent clinical results.

There were some limitations in this systematic review. There was a significant variability in the treatment protocols, follow-up intervals, and measured outcomes between studies. Moreover, it is always a challenge to reproduce future studies related to PRP treatment as there are numerous processing methods and inherent patient individual variability. Even though the number of literature references was limited to three studies, they were all randomized control trials that reported similar results, favoring the combination of PRP and microneedling.

## Conclusion

The use of microneedling to facilitate topical PRP application in AGA cases displayed significantly better results in hair density and hair count than the use of syringe injection. High-quality studies with a more uniform protocol are needed to further confirm these findings.
